# Knockout of reactive astrocyte activating factors slows disease progression in an ALS mouse model

**DOI:** 10.1038/s41467-020-17514-9

**Published:** 2020-07-27

**Authors:** Kevin A. Guttenplan, Maya K. Weigel, Drew I. Adler, Julien Couthouis, Shane A. Liddelow, Aaron D. Gitler, Ben A. Barres

**Affiliations:** 1 Department of Neurobiology, School of Medicine, Stanford University, Stanford, 94305 CA USA; 2 Department of Genetics, School of Medicine, Stanford University, Stanford, 94305 CA USA; 3 Neuroscience Institute, NYU School of Medicine, New York, NY 10016 USA; 4 Department of Neuroscience and Physiology, NYU School of Medicine, New York, NY 10016 USA; 5 Department of Ophthalmology, NYU School of Medicine, New York, NY 10016 USA

**Keywords:** Amyotrophic lateral sclerosis, Astrocyte

## Abstract

Reactive astrocytes have been implicated in the pathogenesis of neurodegenerative diseases, including a non-cell autonomous effect on motor neuron survival in ALS. We previously defined a mechanism by which microglia release three factors, IL-1α, TNFα, and C1q, to induce neurotoxic astrocytes. Here we report that knocking out these three factors markedly extends survival in the *SOD1*^*G93A*^ ALS mouse model, providing evidence for gliosis as a potential ALS therapeutic target.

## Abstract (web summary)

Astrocyte activation may contribute to neurodegenerative disease. Here the authors show that the combined knockout of three factors known to promote astrogliosis, IL-1α, TNFα and C1qa, leads to improved survival in the *SOD1*^*G93A*^ mouse model of ALS.

## Introduction

Amyotrophic lateral sclerosis (ALS) is a devastating neurodegenerative disease caused by a progressive loss of motor neurons, which leads to muscle weakness, paralysis, and eventual death^1^. Beyond motor neurons, numerous studies have implicated glial cells in both the onset and progression of the disease^2–8^. The *SOD1*^*G93A*^ mouse line is the most studied mouse model of ALS, with mice developing many of the pathological hallmarks of patients including distal to proximal motor impairment and eventual motor neuron death^9^. Selectively eliminating the *SOD1*^*G93A*^ transgene from microglia, oligodendrocytes, or astrocytes or performing cell replacement of wild-type microglia or astrocytes into the central nervous system (CNS) of *SOD1*^*G93A*^ animals can slow disease progression and extend lifespan^5,6,10–15^. These experiments have firmly established the contribution of one or more toxic properties of mutant SOD1 within glial cells to drive ALS and have motivated the development of SOD1-lowering therapeutic approaches, which are currently being tested in the clinic^16–18^. Historically, however, it has been difficult to separate the cell autonomous influence of *SOD1*^*G93A*^ transgene expression within glial cells and associated disease phenotypes from the activation of glial cells in response to CNS injury.

We previously determined that microglia activated by neuroinflammatory insults such as lipopolysaccharide (LPS) exposure secrete IL-1α, TNFα, and C1q to induce the conversion of quiescent astrocytes to reactive astrocytes (astrogliosis)^19^. These neuroinflammatory reactive astrocytes lose many of their stereotyped physiological functions and secrete one or more unknown factors that are powerfully toxic to neurons and oligodendrocytes^2,3,19^. This raises the possibility that the established neurotoxicity of rodent astrocytes expressing the SOD1 transgene^2,3^ and human astrocytes derived from patients with ALS^8^ is partially explained by astrocyte reactivity. In general, would preventing the formation of neuroinflammatory reactive astrocytes prove beneficial in patients with ALS?

In this study, we investigate the reactive astrocyte response as a potential therapeutic target in ALS, asking whether neuroinflammatory reactive astrocytes form in ALS as well as whether knocking out the factors that induce these reactive astrocytes is protective in a mouse model of the disease.

## Results

### Knockout of IL-1α, TNFα, and C1q extends survival in an ALS model

**Fig. 1:**
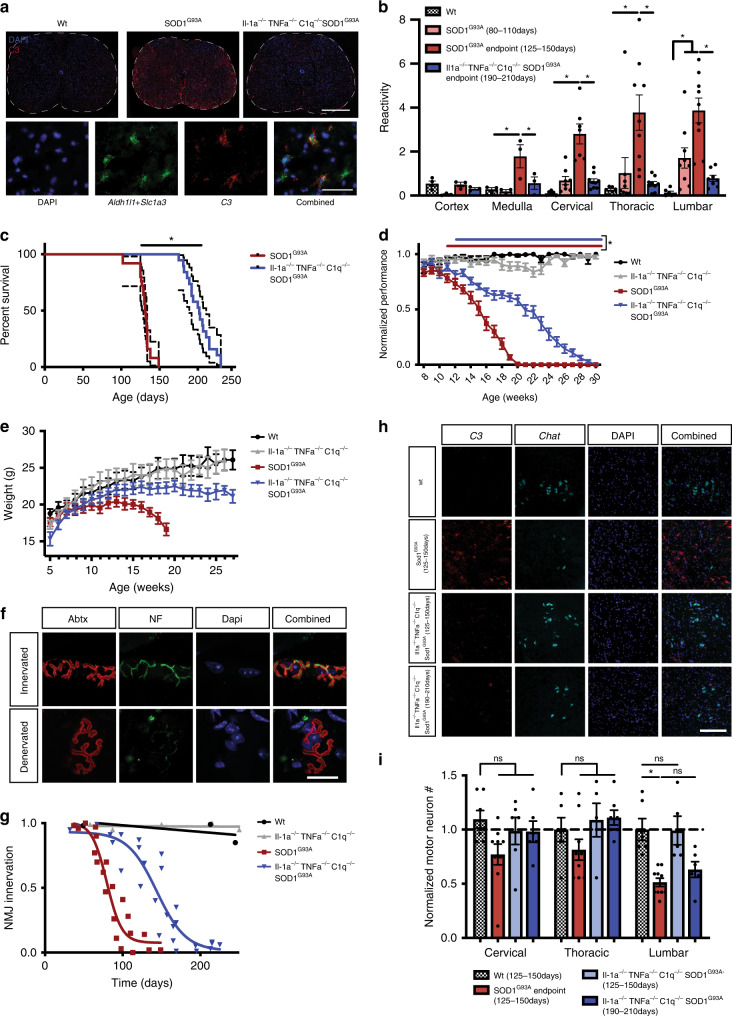
Knockout of IL-1α, TNFα, and C1q prolongs lifespan in the SOD1^G93A^ ALS mouse model. **a** Stitched RNAscope in situ hybridization for the neuroinflammatory reactive astrocyte marker *C3* in the spinal cord of WT, *SOD1*^*G93A*^, and *IL-1α*^−/−^ *TNFα*^−/−^ *C1qa*^−/−^ *SOD1*^*G93A*^ mice (*n* = 3 per genotype; scale bar = 500 μm) and higher magnification examples of *C3*^+^ astrocytes (quantified in Supplementary Fig. 1a; scale bar = 50 μm) **b** Quantification of *C3* in situ hybridization in the cortex, medulla, and spinal cord. (*p < 0.05; two-tailed, unpaired Student’s *t* test, corrected by Holm–Sidak method; mean ± s.e.m.) **c** Survival curve of *SOD1*^*G93A*^ and *IL-1α*^−/−^ *TNFα*^−/−^ *C1qa*^−/−^ *SOD1*^*G93A*^ mice. (*p < 0.05; Gehan–Breslow–Wilcoxon test; dotted lines = 95% confidence interval) **d** Rotarod analysis of motor performance in WT, *SOD1*^*G93A*^, *an IL-1α*^−/−^ *TNFα*^−/−^ *C1qa*^−/−^, and *IL-1α*^−/−^ *TNFα*^−/−^ *C1qa*^−/−^ *SOD1*^*G93A*^ mice. Red bar indicates ages with significantly lower *SOD1*^*G93A*^ performance compared to WT; blue bar indicates ages with significantly lower *IL-1α*^−/−^ *TNFα*^−/−^ *C1qa*^−/−^ *SOD1*^*G93A*^ performance compared to *IL-1α*^−/−^ *TNFα*^−/−^ *C1qa*^−/−^ *(*Mixed-effects model corrected by Tukey method; mean ± s.e.m.) **e** Body weight over time of WT, *SOD1*^*G93A*^, *IL-1α*^−/−^ *TNFα*^−/−^ *C1qa*^−/−^, and *IL-1α*^−/−^ *TNFα*^−/−^ *C1qa*^−/−^ *SOD1*^*G93A*^ mice. (mean ± s.e.m.) **f** Example maximum intensity projections of innervated and denervated neuromuscular junctions. α-Bungarotoxin (Abtx) labels postsynaptic acetylcholine receptors and neurofilament (NF) labels presynaptic motor neuron projections. (quantified in 1 g; scale bar = 20 μm) **g** Quantification of neuromuscular junction innervation. Each point represents 3–6 NMJs from one animal. WT and *IL-1α*^−/−^ *TNFα*^−/−^ *C1qa*^−/−^ curves fit by linear regression and *SOD1*^*G93A*^ and *IL-1α*^−/−^ *TNFα*^−/−^ *C1qa*^−/−^ *SOD1*^*G93A*^ curves fit by sigmoidal interpolation. The IC50s of denervation were 79.6 days for *SOD1*^*G93A*^ and 144 days for *IL-1α*^−/−^ *TNFα*^−/−^ *C1qa*^−/−^ *SOD1*^*G93A*^. **h** Example in situ hybridization against *C3* and *Chat* to quantify motor neuron number in the lateral horn of the spinal cord. (quantified in 1 h; scale bar = 200 μm) **i** Quantification of motor neuron number along the spinal cord in WT, *SOD1*^*G93A*^, and *IL-1α*^−/−^ *TNFα*^−/−^ *C1qa*^−/−^ *SOD1*^*G93A*^ mice (*p < 0.05; two-tailed, unpaired Student’s *t* test, corrected by Holm–Sidak method; mean ± s.e.m.).

In order to determine if neuroinflammatory reactive astrocytes are induced in ALS, we first performed RNAscope in situ hybridization against the neuroinflammatory astrocyte reactivity marker *C3* and saw widespread *C3* upregulation in astrocytes in the spinal cord of *SOD1*^*G93A*^ mice, with the appearance and severity of activation correlating with the onset of symptoms and known progression of neuronal pathology (Fig. 1a, b, Supplementary Fig. 1). To determine if neuroinflammatory reactive astrocytes could be a therapeutic target in ALS, we crossed the *SOD1*^*G93A*^ mouse line to an IL-1α^−/−^ *TNFα*^−/−^ *C1qa*^−/−^ triple knockout mouse line that fails to produce these reactive astrocytes^19^. We assessed the survival of *IL-1α*^−/−^ *TNFα*^−/−^ *C1qa*^−/−^ *SOD1*^*G93A*^ mice compared to strain matched *SOD1*^*G93A*^ mice. Strikingly, *IL-1α*^−/−^ *TNFα*^−/−^ *C1qa*^−/−^ *SOD1*^*G93A*^ mice showed dramatically lower levels of the reactive astrocyte marker *C3* and lived significantly longer than *SOD1*^*G93A*^ mice (an average of 202 vs 131 days, *p* < 0.0001), with an extension of overall lifespan of over 50% (Fig. 1a–c). To our knowledge, this represents one of the longest prolongations of lifespan reported in this model and is especially notable given that individually targeting TNFα in SOD1 mice^20^ or human patients^21^ or targeting Il-1 family members^22,23^, C1q^24^, or the inflammatory astrocyte marker gene *C3* (Supplementary Fig. 2) in various SOD1 mouse models has little or no effect.

The vast majority of ALS cases are sporadic and thus few patients can be identified prior to symptom presentation. Therefore, an ideal therapeutic target may be one that slows the progression of disease rather than preventing disease onset. To determine whether the extension of lifespan in the *IL-1α*^−/−^ *TNFα*^−/−^ *C1qa*^−/−^ *SOD1*^*G93A*^ mice was caused by a delay of disease onset or a slowing of disease progression, we next quantified the impairment of motor strength and coordination in these mice using the accelerating rotarod task. Age of onset of motor impairment was similar in the two models, but the *IL-1α*^−/−^ *TNFα*^−/−^ *C1qa*^−/−^ *SOD1*^*G93A*^ mice showed a much slower deterioration in performance (Fig. 1d), a trajectory of pathogenesis supported by the change in body weight over time (Fig. 1e). These data provide evidence that inhibiting gliosis slows progression rather than delaying disease onset, highlighting the potential of targeting gliosis as a treatment after symptom appearance such as often occurs in sporadic ALS.

Deterioration in motor performance is well correlated with motor neuron denervation of target muscles, a process that precedes motor neuron death^25^. In order to determine if dampening astrogliosis also slows motor neuron denervation, we used confocal fluorescence microscopy to visualize the neuromuscular junctions (NMJs) of the gastrocnemius muscle. We used fluorescently labeled-alpha-bungarotoxin to stain postsynaptic acetylcholine receptors and an antibody against neurofilament protein to stain presynaptic motor neuron inputs (Fig. 1f). We traced maximum projection images of confocal stacks of the postsynaptic and presynaptic regions and quantified the amount of overlap for 3–6 NMJs for each mouse over a range of ages in *SOD1*^*G93A*^ and *IL-1α*^−/−^ *TNFα*^−/−^ *C1qa*^−/−^ *SOD1*^*G93A*^ mice. We modeled denervation over time by sigmoidal interpolation, with 50% motor neuron denervation reached at ~80 days in *SOD1*^*G93A*^ mice, consistent with previous findings (Fig. 1g)^25^. Similar to the impact on overall motor performance, NMJ denervation was slowed in *IL-1α*^−/−^ *TNFα*^−/−^ *C1qa*^−/−^ *SOD1*^*G93A*^ mice by ~65 days, but eventually reached a similar level of pathology at endpoint.

Given the neurotoxic capacity of this reactive astrocyte subtype, we next determined if motor neuron survival was affected in *IL-1α*^−/−^ *TNFα*^−/−^ *C1qa*^−/−^ *SOD1*^*G93A*^ mice. We used RNAscope in situ hybridization to probe for *Chat* and quantify the number of motor neurons at the cervical, thoracic, and lumbar regions of the spinal cord in *SOD1*^G93A^ and *IL-1α*^−/−^ *TNFα*^−/−^ *C1qa*^−/−^ *SOD1*^*G93A*^ mice at both 125–150 days of age (when *SOD1*^*G93A*^ mice reach endpoint) and 190–210 days of age (when *IL-1α*^−/−^ *TNFα*^−/−^ *C1qa*^−/−^ *SOD1*^*G93A*^ mice reach endpoint). As expected, there was an ~50% reduction in motor neuron number in the lumbar spinal cord of *SOD1*^*G93A*^ mice at endpoint, consistent with other studies and methods of motor neuron counting (Fig. 1h–i, Supplementary Fig. 3)^24^. Remarkably, at this same timepoint, there was complete preservation of motor neuron number in *IL-1α*^−/−^ *TNFα*^−/−^ *C1qa*^−/−^ *SOD1*^*G93A*^ mice. However, at later ages when *IL-1α*^−/−^ *TNFα*^−/−^ *C1qa*^−/−^ *SOD1*^*G93A*^ mice reached endpoint, we observed a similar reduction in motor neuron number. Thus, motor neuron death is significantly delayed, but not completely blocked, in the absence of neuroinflammatory reactive astrocytes.

### Cell-autonomous effect of *SOD1*^*G93A*^ on astrocytes and microglia

**Fig. 2:**
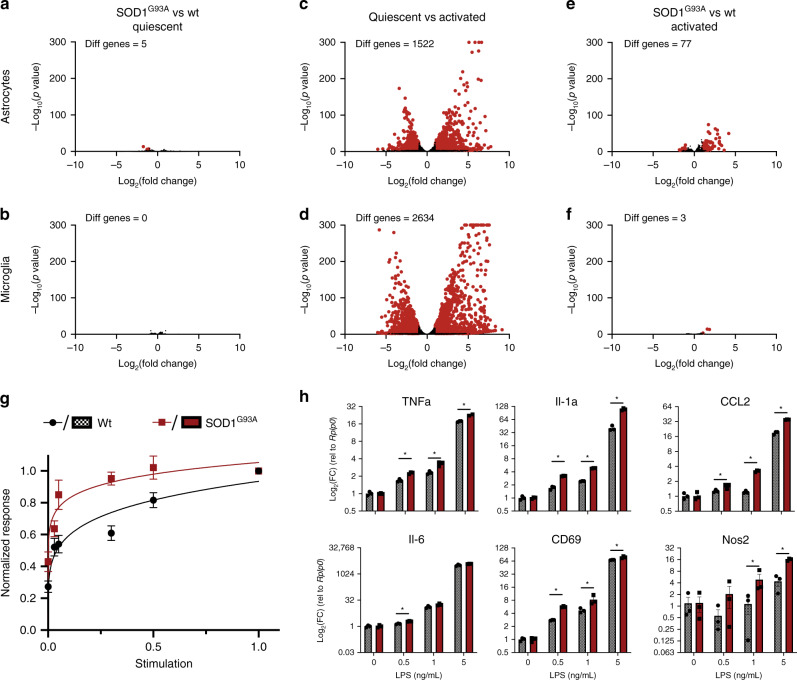
SOD1^G93A^ and WT glia are cell-autonomously similar when quiescent. **a**–**f** Bulk RNA-seq of immunopanned astrocytes and Cd11b MACs purified microglia from WT and *SOD1*^*G93A*^ mice cultured in serum-free conditions. (Red dot indicates significant change with adjusted *p* value < 0.001 and |log_2_(fold change)| > 1) **a**, **b** *SOD1*^*G93A*^ and WT astrocytes and microglia are transcriptomically similar when quiescent in serum-free conditions. **c**, **d** Astrocytes activated by IL-1α (3 ng/mL), TNFα (30 ng/mL), and C1q (400 ng/mL) and microglia activated by LPS (50 ng/mL) change dramatically at the transcriptome level, highlighting the transcriptomic sensitivity of these cells. **e** Fully activated *SOD1*^*G93A*^ and WT astrocytes are largely transcriptomically similar, but show some expression differences associated with cytokine and immune activation. **f** Fully activated *SOD1*^*G93A*^ and WT microglia are transcriptomically similar. **g** Normalized response kinetics of 40 astrocyte reactive marker genes assessed by microfluidics qPCR in response to submaximal doses of IL-1α, TNFα, and C1q. *SOD1*^*G93A*^ astrocytes show larger reactivity responses to smaller insults than WT astrocytes. (error bars represent ± s.e.m.; curves represent nonlinear normalized response curves) **h** Expression of selected microglial activation genes assessed by qPCR in response to submaximal doses of LPS. *SOD1*^*G93A*^ microglia show a slight but significant increase in activation to smaller insults compared to WT microglia. (*p < 0.05 by unpaired, two-tailed Student’s *t* test; Holm–Sidak correction; mean ± s.e.m).

Our results suggest that the microglia-to-astrocyte activation axis powerfully drives disease progression in the *SOD1*^*G93A*^ model of ALS. Many studies have also manipulated *SOD1*^*G93A*^ transgene expression in astrocytes and microglia to modulate disease pathogenesis, and other studies have proposed the protein products of astrocyte reactivity signature genes such as *Lcn2* as mediators of the toxic effect of *SOD1*^*G93A*^-expressing astrocytes their toxic effect on neurons^2,3,26^. Thus, we wanted to determine if there is an interplay between cell-autonomous changes in glial function owing to *SOD1*^*G93A*^ transgene expression and reactive astrogliosis. We isolated primary astrocytes and microglia from WT or *SOD1*^*G93A*^ mice by immunopanning^27^ or MACS isolation^28,29^ and cultured them in serum-free conditions^30^. Unexpectedly, WT and *SOD1*^*G93A*^ astrocytes and microglia were strikingly similar when quiescent (Fig. 2a, b, Supplementary Fig. 4; http://www.gliaseq.com). This stands in stark contrast to the overall transcriptomic excitability of these cells, which respond dramatically to Il-1α, TNFα, and C1q in the case of astrocytes and LPS in the case of microglia (Fig. 2c, d). Thus, *SOD1*^*G93A*^ astrocytes and microglia appear remarkably similar to WT cells when quiescent.

Given their role in exacerbating disease progression, *SOD1*^*G93A*^ glial cells may show differences in activation following insults, such as would occur after the neuron-intrinsic initiation of pathology in ALS. To test this, we first performed RNA-seq on WT and *SOD1*^*G93A*^ astrocytes and microglia exposed to maximally stimulating doses of Il-1α, TNFα, and C1q in the case of astrocytes and LPS in the case of microglia. While maximally activated *SOD1*^*G93A*^ and WT microglia were virtually identical, activated *SOD1*^*G93A*^ astrocytes upregulated many more genes than WT astrocytes (Fig. 2e, f, Supplementary Fig. 4) with GO terms related to immune activation (Supplementary Table 1). In addition to changing in response to large insults, expression of mutant *SOD1*^*G93A*^ may also more subtly influence how activated astrocytes and microglia respond to subthreshold stimulation. To test this hypothesis in astrocytes, we used a microfluidics qPCR panel that we have previously used to characterize astrocyte reactivity in vivo and in vitro^19^. We first found that WT and *SOD1*^*G93A*^ astrocytes responded identically in the microfluidics panel when maximally stimulated by a full dose of Il-1α, TNFα, and C1q (Supplementary Fig. 5). Exposing astrocytes to submaximal doses of the activating factors Il-1α, TNFα, and C1q and normalizing each gene’s expression to its response to maximal activation, *SOD1*^*G93A*^ astrocytes reached high levels of reactivity at much lower doses than WT (Fig. 2h). This precocious response to lower doses of IL-1α, TNFα, and C1q suggests that mutant *SOD1*^*G93A*^ may make astrocytes primed to respond to subtle insults that would otherwise not mount a sufficient response to alter neuronal viability or function. We next investigated the microglial response from WT and *SOD1*^*G93A*^ cells and saw a similar response. When assessing the change in expression of known microglial activation genes by qPCR, *SOD1*^*G93A*^ microglia showed larger increases in reactivity than WT microglia in response to low doses of LPS (Fig. 2h). These results suggest that modulating *SOD1*^*G93A*^ expression in astrocytes and microglia may subtly change their propensity for activation in early stages of the disease, and changes in gliosis may partially explain the effects seen in vivo from modulating levels of mutant *SOD1* expression in these cells. Further, these results align with previous studies investigating the activation of glial cells at early timepoints in mouse models of ALS^31^, but also highlight that that these changes in astrocytes are cell autonomous. Finally, the increase in neuroinflammatory astrocyte reactivity in aging combined with the effect of mutations such as *SOD1*^*G93A*^ on the propensity for glial activation could partially explain the role of aging as a risk factor for ALS and other neurodegenerative diseases^32,33^.

### C3^+^ reactive astrocytes appear in familial and sporadic ALS

**Fig. 3:**
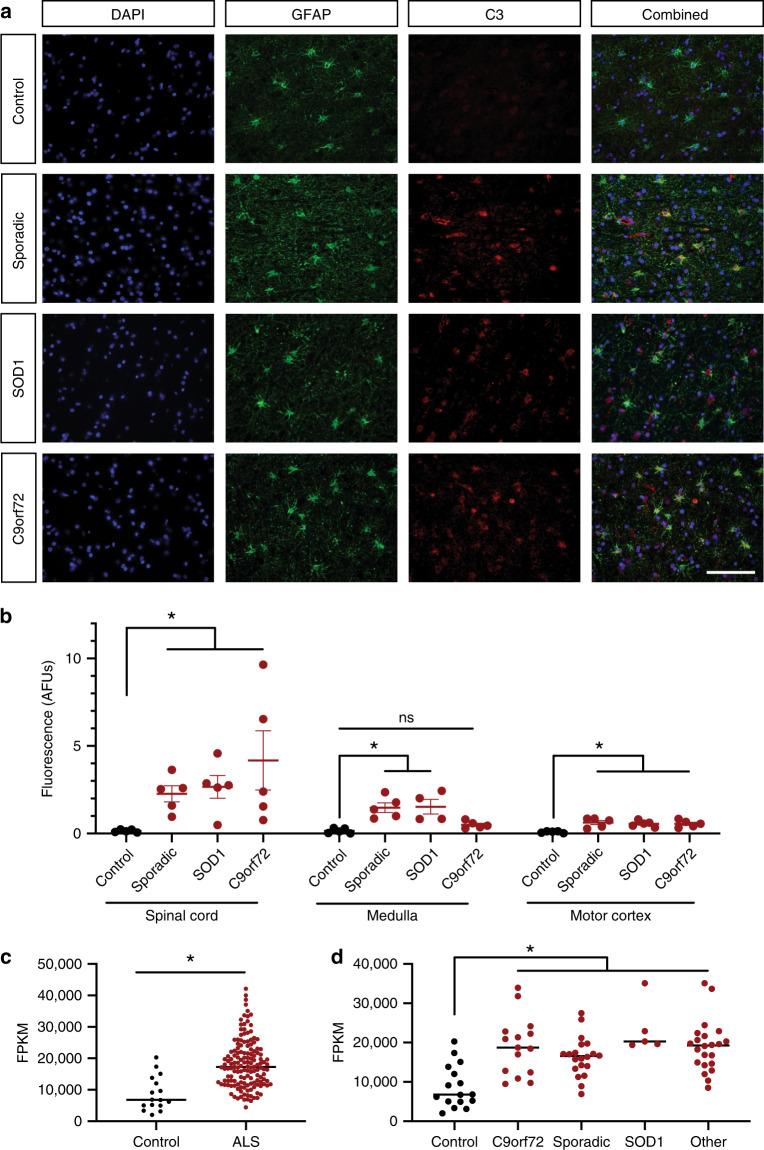
Neuroinflammatory astrogliosis is a common pathology in human ALS. **a** Example images of cortical samples from patients with sporadic, SOD1, and C9orf72 associated ALS as well as nonneurological controls stained with DAPI for nuclei, GFAP for astrocytes, and C3 as a marker for neuroinflammatory astrocyte activation. (quantified in **b**; scale bar = 100 µm) **b** Quantification of C3 immunoreactivity within GFAP^+^ astrocytes in the spinal cord, medulla, and cortex. There was significantly more C3 staining within astrocytes in all subtypes of ALS compared to controls in the spinal cord, medulla, and cortex except for samples of the medulla from C9orf72 associated ALS patients. (*p < 0.05 by Kruskal–Wallis nonparametric test corrected for multiple comparisons using the Dunn method; mean ± s.e.m.) **c** Bulk RNA-seq *C3* expression from the spinal cord of ALS patients vs control (*p < 0.05 by unpaired, two-tailed Student’s *t* test; mean). **d** Subsampling of data in **c** by identified ALS subtype (other = other genetic cause; *p < 0.05; two-tailed, unpaired Student’s *t* test, corrected by Dunnett method; mean).

Reactive gliosis appears to powerfully influence disease pathogenesis in the *SOD1*^*G93A*^ mouse model of ALS, but do astrocytes respond similarly in human ALS? Importantly, while most mouse models of ALS rely on the genetics of familial ALS, most ALS cases are sporadic and thus broadly beneficial therapeutics should target mechanisms that are common to sporadic as well as familial forms of the disease. To determine if neuroinflammatory astrocyte activation appears in sporadic as well as familial ALS, we preformed GFAP and C3 staining on tissue sections of the spinal cord, medulla, and motor cortex from human patients with sporadic ALS, and familial cases with either *C9ORF72* or *SOD1* mutations, and age-matched nonneurological controls (Supplementary Table 2, Supplementary Fig. 6). Quantifying the fluorescence intensity of C3 within astrocytes, we saw a significant increase in astrogliosis in the spinal cords and motor cortices of the sporadic ALS cases and the *C9ORF72* and SOD1-ALS cases, as well as an increase in C3 levels in the medulla of sporadic and SOD1-ALS patients (Fig. 3a, b), changes that accompanied traditional astrocyte hypertrophy (Supplementary Fig. 7). To validate these increases in C3 protein in astrocytes, we also assessed *C3* expression from bulk RNA-seq acquired from the spinal cord of a large series of ALS patients and nonneurological controls (16 control patients, 148 ALS patients). In line with our immunohistochemistry results, there was a significant increase in *C3* mRNA expression in the spinal cord of ALS patients (Fig. 3c). Further, subsampling this data for cases that were definitively defined as sporadic, *C9ORF72*, *SOD1*, or other genetically inherited forms of ALS, we found the increase in *C3* expression was common to every ALS subtype (Fig. 3d).

## Discussion

Together, these experiments provide evidence that neuroinflammatory astrogliosis is a common pathology in human ALS that powerfully controls disease progression in a mouse model of the disease. The marked prolongation of lifespan achieved by eliminating Il-1α, TNFα, and C1q suggests that the modulation of gliosis may prove a powerful avenue for novel therapeutics. The use of global knockouts of these genes does not preclude the contribution of mechanisms outside the CNS. But any such peripheral contributions might actually facilitate therapeutic intervention because a partial effect may be achievable without requiring inhibitors of this pathway to cross the blood–brain barrier. Further, since the dampening of astrogliosis modulates the systemic response to neuronal damage, therapeutic interventions targeting gliosis could be used in sporadic patients after disease onset as well as in conjunction with antisense oligonucleotide therapies targeting causative familial mutations such as *SOD1* and *C9ORF72*^16–18^. The delay in disease progression afforded by this treatment may buy time for such antisense therapeutics targeting mutant genes to take effect within motor neurons. Thus, a combination of therapies targeting multiple mechanisms (e.g., motor neurons and reactive astrocytes) might be more efficacious than either individually. Since neuroinflammatory astrocytes seem to be prevalent in other neurodegenerative diseases, including Alzheimer’s disease^34,35^, Parkinson’s disease^36^, glaucoma^37^, and aging^32^, our findings also highlight gliosis as a therapeutic target for neurodegeneration more broadly.

## Methods

### Animals

All animal procedures were conducted in accordance with guidelines from the National Institute of Health and Stanford University’s Administrative Panel on Laboratory Animal Care. All mice were housed with food and water available ad libitum in a 12-h light/dark environment at 68–72 **°**F and 30–70% humidity. *IL-1α*^−/−^ *TNFα*^−/−^ *C1qa*^−/−^animals were developed in house^19^. *SOD1*^*G93A*^ were obtained from Jax (002726) and bred into either C57BL/6J (Jax, 000664), *IL-1α*^−/−^ *TNFα*^−/−^ *C1qa*^−/−^, or *C3*^−/−^ (Jax, strain 003641) lines in house for eight generations before testing. Animals were randomly assigned and tested blind to both experimental condition and genotype.

### RNAscope in situ hybridization and quantification

RNAscope fluorescent in situ hybridization was performed on fresh-frozen tissue. After sacrifice, brains were removed by dissection and spinal cords were removed by hydraulic extrusion into ice-cold PBS. Tissue was embedded in OCT compound (Tissue-Tek) and 14 μm tissue sections were prepared and immediately frozen at −80 °C. Multiplex RNAscope was performed according to manufacturer’s protocol against the targets *C3*, *Aldh1l1*, *Slc1a3*, and *Cx3cr1*. In order to achieve complete and robust labeling of astrocytes, we imaged probes against both astrocyte markers *Aldh1l1* and *Slc1a3* in the same channel, denoted by *Aldh1l1* + *Slc1a3* in Fig. 1 and Supplementary Fig. 1.

RNAscope in situ hybridization is nonlinearly amplified and thus intensity cannot be used to measure expression. Instead, images of *C3* in situ hybridization were randomly taken from the ventral horn and thresholded in ImageJ using the Moments parameter set, all blind to genotype and age (ImageJ v2.0.0 build 269a0ad53f). The percent of area covered by this thresholded signal was then quantified in the gray matter of the spinal cord (with white matter excluded by DAPI signal) and this value recorded as reactivity. The number of Chat^+^ motor neurons in the ventral horn was manually counted for one tissue section per spinal cord region per animal, blinded to genotype and age. The region of the spinal cord (cervical, thoracic, lumbar) for each section was validated by comparison to an anatomical atlas.

### Cresyl violet staining

Fresh-frozen tissue sections were first dehydrated in 1:1 ethanol/chloroform overnight. Slides were then rehydrated in 100% ethanol and 95% ethanol for 5 min each. 0.1% Cresyl violet stain was then prepared fresh, with 0.5% glacial acetic acid added before solution filtering immediately prior to use. Slides were stained in 0.1% Cresyl violet solution for 15 min at room temperature (RT) before being dipped in distilled water and then differentiated in 95% ethanol for 60 s. Slides were then dehydrated in 100% alcohol for 5 min and then cleared 2× in xylene for 5 min each. Slides were then mounted in limonene mounting media and motor neurons counted by the experimenter blind to condition.

### SOD1^G93A^ behavioral and weight analysis

*SOD1*^*G93A*^ studies were conducted in accordance with protocols approved by the Administrative Panel of Laboratory Animal Care of Stanford University. All *SOD1*^*G93A*^ mice were given DietGel Boost soft food (ClearH2O) and HydroGel (ClearH2O) from P100 until euthanasia to ensure the animals were adequately fed and hydrated. Human euthanasia point was used instead of natural death to ensure animal welfare and was defined as the inability of the mice to right themselves within 10 s after being placed on their backs or a body condition score < 2.

Rotarod was used to assess motor coordination, strength and balance over the course of disease progression. Mice were first trained on the rotarod for three sessions 1 week prior to initial analysis at 8 weeks of age. For testing, a 4–40 program was used in which the speed gradually increases from 4 to 40 rpm over 5 min. The animals had three attempts each testing session with maximum experimental run of 360 s. The largest latency to fall of three trials was recorded each week until humane endpoint was reached (or equivalent age for littermate controls). Each animal’s performance was normalized to its longest latency over the entire experiment.

### Neuromuscular junction innervation analysis

The gastrocnemius muscle of the rear leg was acquired by blunt dissection and fixed for 12–24 h in 1% paraformaldehyde (note that fixation in 4% paraformaldehyde disrupted later staining for our assays). Fixed muscles were transitioned to 30% sucrose for 24 h or until fully cryoprotected. Muscles were then embedded in OCT compound (Tissue-Tek) and 14 μm sections were acquired by a Leica cryostat.

Muscle section were permeabilized with 0.1% Triton X for 10 min followed by blocking with 50% normal goat serum (NGS) in PBS for 1 h. Sections were stained with a rabbit anti neurofilament heavy polypeptide antibody (1:500, abcam: ab8135) for 3 h at RT or overnight at 4 °C. After four washes with PBS, sections were then stained with an appropriate secondary antibody as well as Alexa Fluor 647 conjugated α-bungarotoxin (Invitrogen, B35450) for 1–2 h at RT. After four washes with PBS, slides were mounted with VECTASHIELD Antifade Mounting Medium with DAPI (Vector Labs, H-1200).

Slides were imaged on a Zeiss LSM710 Confocal Microscope using Zen 2012 v. 14.09.201 software. NMJs were selected at random using the α-bungarotoxin signal and a confocal stack taken at ×63 through the depth of the NMJ. Maximum projections images were then skeletonized blind and the linear extent of the α-bungarotoxin signal filled by neurofilament quantified as a fraction. *SOD1*^*G93A*^ and *IL-1α*^−/−^ *TNFα*^−/−^ *C1qa*^−/−^ *SOD1*^*G93A*^ data were fit by sigmoidal interpolation and WT and *IL-1α*^−/−^ *TNFα*^−/−^ *C1qa*^−/−^ data fit by linear regression.

### Immunopanning and cell culture

Astrocytes were purified by immunopanning from P5 WT mouse forebrains and cultured as previously described^27^. Cortices were blunt dissected and enzymatically digested using papain at 37 °C and 10% CO_2_. Tissue was then mechanically triturated with a 5 mL serological pipette at RT to generate a single-cell suspension. The suspension was filtered in a 70 µm nitex filter and negatively panned for microglia (CD45, BD Pharmingen 554875), endothelial cells (BSL I, Vector Labs L-1100), and oligodendrocyte lineage cells (O4 hybridoma, in house) followed by positive panning for astrocytes (HepaCAM, R&D Systems MAB4108). Astrocytes were removed from the final positive selection plate by brief digestion with 0.025% trypsin and plated on poly-d-lysine coated tissue culture plates. Astrocytes were cultured in defined, serum-free medium containing 50% neurobasal, 50% DMEM, 100 U/mL penicillin, 100 μg/mL streptomycin, 1 mM sodium pyruvate, 292 μg/mL l-glutamine, 1× SATO, 5 μg/mL of N-acetyl cysteine, and 5 ng/mL HBEGF (Peptrotech, 100–47).

Microglia were purified by magnetic activated cell separation as described previously, with all steps occurring on ice or in a 4 °C cold room^28^. Briefly, p7–p10 mice were euthanized by CO_2_ and transcardially perfused with dPBS using a syringe to remove circulating macrophages and monocytes. Whole brains were then cut into ~1 mm^3^ pieces and dounced in ice-cold Medium A containing 15 mM HEPES and 0.5% glucose in 1x HBSS and spun at 500 × *g* for 5 min to remove dead cells and debris. The cell pellet was resuspended in MACS Buffer containing 2 mM EDTA and 0.5% BSA (w/v, Sigma, A4161) in 1x PBS containing myelin removal beads (Miltenyi, 130-096-733). After incubating for 15 min at 4 °C, the cell suspension was diluted with MACS Buffer and centrifuged at 500 × *g* for 5 min to remove unbound myelin removal beads. The pellet was resuspended in MACS Buffer and passed through an LD MACS column (Miltenyi, 130-042-901) to collect the flow-through of unbound cells. Cells were pelleted for 30 s at 10k × *g* and resuspend in MACS Buffer containing CD11b beads (Miltenyi, 130-049-601). The suspension was incubated for 15 min at 4 °C, diluted, and pelleted for 30 s at 10k × *g* to remove unbound Cd11b beads. The pellet was then resuspended in MACS Buffer and passed through an LS MACS column (Miltenyi, 130-042-401). Bound microglia were eluted from the MACs column and plated on poly-d-lysine coated tissue cultures plates. Microglia were cultured in a minimal, serum-free media consisting of DMEM/F12 (Gibco, 21041025) supplemented with 100 units/mL penicillin, 100 μg/mL streptomycin, 2 mM glutamine, 5 μg/mL N-acetyl cysteine, 100 μg/mL apo-transferrin, 100 ng/mL sodium selenite, TGF-β2 (2 ng/mL, Peprotech), murine IL-34 (100 ng/mL, R&D Systems), ovine wool cholesterol (1.5 μg/mL, Avanti Polar Lipids), and heparan sulfate (1 μg/mL, Galen Laboratory Supplies).

### RNA-sequencing

Isolated astrocyte and microglia RNA samples were first assayed on an Agilent 2100 Bioanalyzer System to quantify RNA quality and total RNA abundance. mRNA libraries were then prepared for Illumina paired-end sequencing using the Agilent SureSelect Strand Specific RNA-Seq Library Preparation kit (G9691B) on the Agilent Bravo Automated Liquid Handling Platform. Libraries were sequenced on an Illumina HiSeq 4000. Alignment of RNA-sequencing reads to the mouse mm10 reference genome and transcriptome was performed using STAR v2.7.3a^38^ following ENCODE standard options, read counts were generated using rsem, and differential expression analysis was performed in R v3.6.1 using the DESeq2 package v1.38.0^39^ (detailed pipeline v2.0.1 and options available on https://github.com/emc2cube/Bioinformatics/).

Bulk RNA-seq FastQ files from ALS spinal cord samples were acquired through Target ALS (http://www.targetals.org/research/resources-for-scientists/resource-genomic-data-sets/) and analyzed using the above pipeline, using the hg38 human reference genome and transcriptome for alignment.

### Microfluidics qPCR

Total RNA was extracted using the RNeasy Plus Kit (Qiagen) and cDNA synthesis performed using the High-Capacity RNA-to-cDNA Kit (Applied Biosystems) according to manufacturer protocol. Overall, 1.25 μL of each cDNA sample was preamplified using 2.5 μL of 2× Taqman preamplification master mix (Applied Biosystems) and 1.25 μL of the primer pool (0.2 pmol of each primer per μL; primer pool as previously described^19^). Preamplification was performed with a 95 °C denaturation step for 10 min followed by 14 cycles of 95 °C for 15 s and 60 °C for 4 min. Products were diluted 5× in TE Buffer (Teknova), and 5 μL of a sample mix containing preamplified cDNA and amplification Master mix (20 mM MgCl_2_, 10 mM dNTPs, FastStart Taq polymerase, DNA-binding dye loading reagent, 50× ROX, 20× Evagreen) was loaded into each sample inlet of a 96.96 Dynamic Array chip (Fluidigm) and 5 μL from an assay mix containing DNA-assay loading reagent, as well as forward and reverse primers (10 pmol/μL) was loaded into each detector inlet. The chip was then loaded and mixed in the NanoFlexTM 4-IFC Controller (Fluidigm). Next, the chip was processed in the BioMark Real-Time PCR System (Fluidigm) using a cycling program of 10 min at 95 °C followed by 40 cycles of 95 °C for 15 s, 60 °C for 30 s and 72 °C for 30 s, followed by a melting curve. Cycle of quantification (Cq) data were collected using BioMark Data Collection Software 2.1.1 build 20090519.0926 (Fluidigm). Corrections were made for differences in input RNA using the geometric mean of three reference genes Aldh1l1, Gapdh and Rplp0. Data preprocessing and analysis was completed using Fluidigm Melting Curve Analysis Software 1.1.0 build 20100514.1234 (Fluidigm) and Real-time PCR Analysis Software 2.1.1 build 20090521.1135 (Fluidigm) to determine valid PCR reactions. Invalid reactions were removed from later analysis. See Supplementary Table 5 for a list of all primers used in microfluidics qPCR experiments.

### Quantitative PCR

Total RNA was extracted using the RNeasy Plus Kit (Qiagen) and cDNA synthesis performed using the High-Capacity RNA-to-cDNA Kit (Applied Biosystems) according to manufacturer protocol. qPCR was performed using Fast SYBR Green (Applied Biosystems) with a cycling program of 95 °C for 20 s followed by 40 cycles of 95 °C for 3 s and 60 °C for 30 s, and ending with a melting curve. Relative mRNA expression was normalized to *Rplp0*. Primers used were: tnf: fwd gatcggtccccaaagggatg rev tgtgagggtctgggccatag; il1a: fwd cgcttgagtcggcaaagaaat rev tggcagaactgtagtcttcgt; ccl2: fwd agctgtagtttttgtcaccaagc rev gtgctgaagaccttagggca; il6: fwd tcctctctgcaagagacttcc rev ttgtgaagtagggaaggccg; cd69: fwd tccgtggaccacttgagagt rev atactggtgccatggtcctt; nos2: fwd tcctggacattacgacccct rev ctctgagggctgacacaagg.

### Staining of human tissue

Paraffin embedded postmortem brain sections from the motor cortex, lumbar or cervical spinal cord, and medulla were obtained from the University of Pennsylvania Institute on Aging. The Federalwide Assurance (FWA) for NYU Langone (#00004952) was approved by the Office for Human Research Protections at the U.S. Department of Health and Human Services (DHHS). All tissue was de-identified and provided without link to identifiable information from an IRB-approved tissue repository that acquired informed consent. Control individuals were pathologically deemed to have “unremarkable brains”, while ALS patients carried SOD1 mutations, C9orf72 mutations, or sporadic etiologies. The paraffin was removed with 2 xylene washes for 5 min each. Sections were rehydrated sequentially twice in 100% EtOH, once in 95% EtOH, once in 70% EtOH, and once in 1x tris-buffered saline (TBS, pH 7.6) for 5 min each. Sections were then submerged in M6 buffer (2.1% citric acid monohydrate, 2.94% tris-sodium citrate in dH2O, pH 6), microwaved until bubbling, and placed in a 98 °C water bath for 10 min for antigen retrieval. Sections were then washed three times in 1xTBS for 5 min each and blocked for 30 min in 10% NGS in 1xTBS at RT. Subsequently sections were incubated in primary stains rabbit anti-C3d 1:600 (DAKO A0063) and mouse anti-GFAP 1:400 (Sigma G3893) overnight at 4 °C in a humidity chamber. Primary stained sections were then washed three times in 1xTBS and incubated in secondary stains Alexa-594 goat anti-rabbit 1:500 (ThermoFisher R37117) and Alexa-647 goat anti-mouse 1:1000 (ThermoFisher A-21235) for 30 min at RT covered from light. Sections were washed three times for 5 min in 1xTBS and incubated in DAPI stain 1:10,000 (ThermoFisher D1306) for 5 min. Sections were washed five times in 1xTBS for 5 min each and incubated for 1–2 min in TrueBlack Lipofuscin (Biotium 23007) 1:20 diluted in 100% EtOH. Sections were washed three times in 1xTBS and mounted in Fluoromount-G (SouthernBiotech 0100–01).

Section staining did not afford the experimenters the ability to definitively delineate the cell body and processes of individual astrocytes by GFAP staining in all conditions. To measure hypertrophy, images of GFAP staining were thresholded in ImageJ using the Moments parameter set, blind to genotype and age. The percent of area covered by this thresholded signal was then quantified and this value recorded as a measure of total cell body and process size.

### Reporting summary

Further information on research design is available in the Nature Research Reporting Summary linked to this article.

## Supplementary information

Supplementary Information

Peer Review File

Reporting Summary

## Data Availability

Source data are available as a Source Data file. In vitro RNA-seq data available at website www.gliaseq.com. Raw in vitro RNA-seq data available at https://www.ncbi.nlm.nih.gov/geo/query/acc.cgi?acc=GSE143598. All other data are available from the corresponding authors upon reasonable request. See Supplementary Table 3 for number of biological replicates used in each experiment and Supplementary Table 4 for exact *p* values for statistical comparisons. All statistics performed using Prism v 8.2.1.

## Source data

Source Data

## Code availability

RNA-sequencing pipeline code available at https://github.com/emc2cube/Bioinformatics/.

## References

[1] Taylor JP, Brown RH, Cleveland DW (2016). Decoding ALS: from genes to mechanism. Nature.

[2] Nagai M (2007). Astrocytes expressing ALS-linked mutated SOD1 release factors selectively toxic to motor neurons. Nat. Neurosci..

[3] Giorgio F, Carrasco MA, Siao MC, Maniatis T, Eggan K (2007). Non–cell autonomous effect of glia on motor neurons in an embryonic stem cell–based ALS model. Nat. Neurosci..

[4] Ferraiuolo L (2016). Oligodendrocytes contribute to motor neuron death in ALS via SOD1-dependent mechanism. Proc. Natl Acad. Sci. USA.

[5] Beers DR (2006). Wild-type microglia extend survival in PU.1 knockout mice with familial amyotrophic lateral sclerosis. Proc. Natl Acad. Sci..

[6] Clement AM (2003). Wild-type nonneuronal cells extend survival of SOD1 mutant motor neurons in ALS mice. Science.

[7] Frakes AE (2014). Microglia induce motor neuron death via the classical NF-κB pathway in amyotrophic lateral sclerosis. Neuron.

[8] Haidet-Phillips AM (2011). Astrocytes from familial and sporadic ALS patients are toxic to motor neurons. Nat. Biotechnol..

[9] Gurney M (1994). Motor neuron degeneration in mice that express a human Cu,Zn superoxide dismutase mutation. Science.

[10] Boillée S (2006). Onset and progression in inherited ALS determined by motor neurons and microglia. Science.

[11] Wang L, Gutmann DH, Roos RP (2011). Astrocyte loss of mutant SOD1 delays ALS disease onset and progression in G85R transgenic mice. Hum. Mol. Genet.

[12] Wang L, Sharma K, Grisotti G, Roos RP (2009). The effect of mutant SOD1 dismutase activity on non-cell autonomous degeneration in familial amyotrophic lateral sclerosis. Neurobiol. Dis..

[13] Yamanaka K (2008). Mutant SOD1 in cell types other than motor neurons and oligodendrocytes accelerates onset of disease in ALS mice. Proc. Natl Acad. Sci..

[14] Kang SH (2013). Degeneration and impaired regeneration of gray matter oligodendrocytes in amyotrophic lateral sclerosis. Nat. Neurosci..

[15] Ilieva H, Polymenidou M, Cleveland DW (2009). Non-cell autonomous toxicity in neurodegenerative disorders: ALS and beyond. J. Cell Biol..

[16] McCampbell A (2018). Antisense oligonucleotides extend survival and reverse decrement in muscle response in ALS models. J. Clin. Investig..

[17] Miller TM (2013). An antisense oligonucleotide against SOD1 delivered intrathecally for patients with SOD1 familial amyotrophic lateral sclerosis: a phase 1, randomised, first-in-man study. Lancet Neurol..

[18] Smith RA (2006). Antisense oligonucleotide therapy for neurodegenerative disease. J. Clin. Investig.

[19] Liddelow SA (2017). Neurotoxic reactive astrocytes are induced by activated microglia. Nature.

[20] Gowing G, Dequen F, Soucy G, Julien J-P (2006). Absence of tumor necrosis factor- does not affect motor neuron disease caused by superoxide dismutase 1 mutations. J. Neurosci..

[21] Petitpain N (2019). Is TNF inhibitor exposure a risk factor for amyotrophic lateral sclerosis?. Fundam Clin. Pharm..

[22] Han Y, Ripley B, Serada S, Naka T, Fujimoto M (2016). Interleukin-6 deficiency does not affect motor neuron disease caused by superoxide dismutase 1 mutation. PLoS ONE.

[23] Nguyen MD, Julien J-P, Rivest S (2001). Induction of proinflammatory molecules in mice with amyotrophic lateral sclerosis: no requirement for proapoptotic interleukin-1beta in neurodegeneration. Ann. Neurol..

[24] Lobsiger CS (2013). C1q induction and global complement pathway activation do not contribute to ALS toxicity in mutant SOD1 mice. Proc. Natl Acad. Sci..

[25] Fischer LR (2004). Amyotrophic lateral sclerosis is a distal axonopathy: evidence in mice and man. Exp. Neurol..

[26] Bi F (2013). Reactive astrocytes secrete lcn2 to promote neuron death. Proc. Natl Acad. Sci. USA.

[27] Foo LC (2011). Development of a method for the purification and culture of rodent astrocytes. Neuron.

[28] Bennett ML (2016). New tools for studying microglia in the mouse and human CNS. Proc. Natl Acad. Sci..

[29] Bohlen CJ (2017). Diverse requirements for microglial survival, specification, and function revealed by defined-medium cultures. Neuron.

[30] Guttenplan KA, Liddelow SA (2018). Astrocytes and microglia: models and tools. J. Exp. Med..

[31] Sun S (2015). Translational profiling identifies a cascade of damage initiated in motor neurons and spreading to glia in mutant SOD1-mediated ALS. Proc. Natl Acad. Sci..

[32] Clarke LE (2018). Normal aging induces A1-like astrocyte reactivity. Proc. Natl Acad. Sci..

[33] Boisvert MM, Erikson GA, Shokhirev MN, Allen NJ (2018). The aging astrocyte transcriptome from multiple regions of the mouse brain. Cell Rep..

[34] Joshi AU (2019). Fragmented mitochondria released from microglia trigger A1 astrocytic response and propagate inflammatory neurodegeneration. Nat. Neurosci..

[35] Shi Y (2017). ApoE4 markedly exacerbates tau-mediated neurodegeneration in a mouse model of tauopathy. Nature.

[36] Yun SP (2018). Block of A1 astrocyte conversion by microglia is neuroprotective in models of Parkinson’s disease. Nat. Med..

[37] Guttenplan KA (2020). Neurotoxic reactive astrocytes drive neuronal death after retinal injury. Cell Rep..

[38] Dobin A (2012). STAR: ultrafast universal RNA-seq aligner. Bioinform. Oxf. Engl..

[39] Love MI, Huber W, Anders S (2014). Moderated estimation of fold change and dispersion for RNA-seq data with DESeq2. Genome Biol..

