# Supplementary material for: Knockout of reactive astrocyte activating factors slows disease progression in an ALS mouse model
Source: Nat Commun. 2020 Jul 27;11:3753. doi: 10.1038/s41467-020-17514-9 (PMC7385161; doi:10.1038/s41467-020-17514-9)
Supplement: Supplementary file 1 — Supplementary Information [file 41467_2020_17514_MOESM1_ESM.pdf]

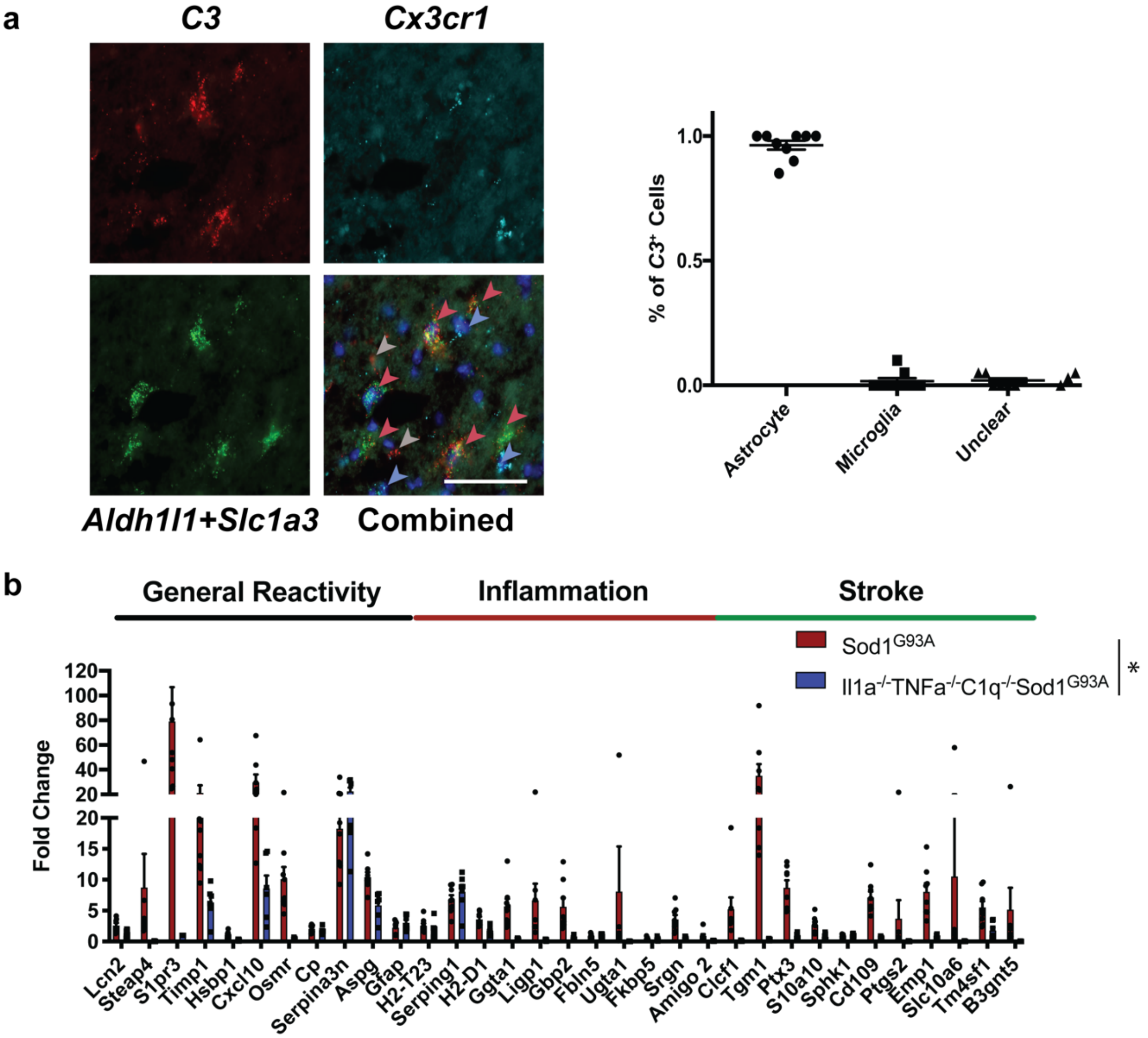

Supplementary Figure 1. Astrocytes in the Sod1^{G93A} spinal cord show broad reactivity. a. We assessed the cell type specificity of *C3* RNAscope *in situ* hybridization signal in the spinal cord by probing for *Aldh1l1+Slc1a3* for astrocytes and *Cx3cr1* for myeloid cells. Example image shows cells labeled as *C3*$^+$ astrocytes (red arrowhead), myeloid cells (blue arrowhead), and *C3*$^+$ cells of unknown cell-type (grey arrowhead). (scale bar = 50 µm) Quantification shows that the vast majority of *C3* signal is associated with astrocytes. (lines represent mean ± SEM) **b.** We performed microfluidics qPCR on whole spinal cords from endpoint *Sod1^{G93A}* and *IL-1α$^{-/-}$ TNFα$^{-/-}$ C1q$^{-/-}$ Sod1^{G93A}* mice using our panel of genes that characterize astrocyte reactivity. *IL-1α$^{-/-}$ TNFα$^{-/-}$ C1q$^{-/-}$ Sod1^{G93A}* mice show a broadly damped reactivity response compared to *Sod1^{G93A} mice* (fold change relative to untreated control; error bars represent ± SEM; * = p<0.0001 by paired, two-tailed paired t-test).

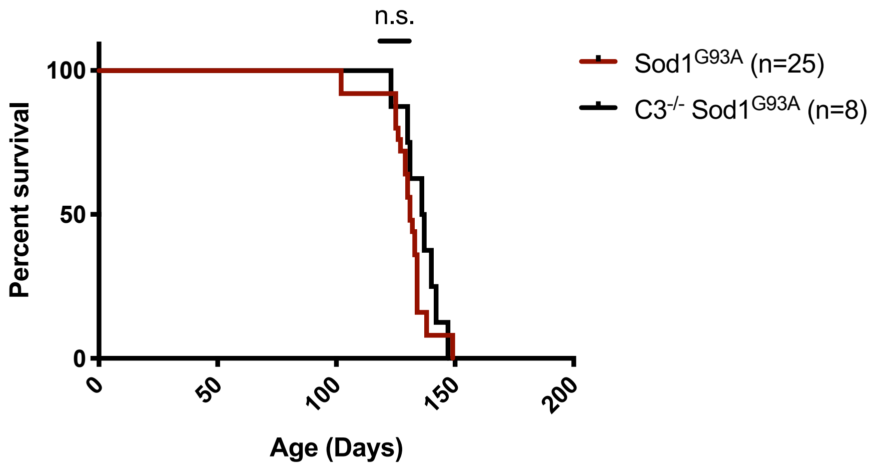

Supplementary Figure 2. Knocking out the neuroinflammatory astrocyte marker *C3* has no effect on lifespan in the Sod1^{G93A} mouse model of ALS. Survival curves of C3$^{-/-}$Sod1^{G93A} mice compared to Sod1^{G93A} (Sod1^{G93A} data also plotted in Figure 1c; n.s = $p > 0.05$; Gehan-Breslow-Wilcoxon test)

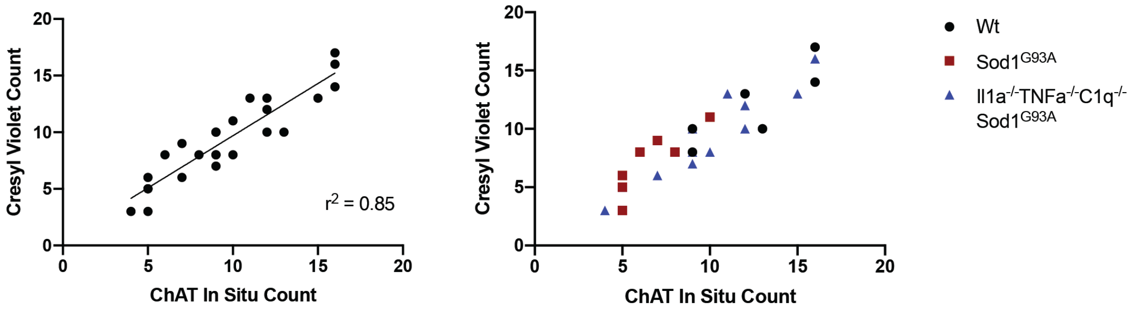

Supplementary Figure 3. Comparison of motor neuron counts in selected images by Cresyl violet staining vs RNAscope *in situ* hybridization again *Chat*. Comparison of ChAT *in situ* hybridization and Cresyl Violet methods of motor neuron quantification show similar results in a variety of genotypes.

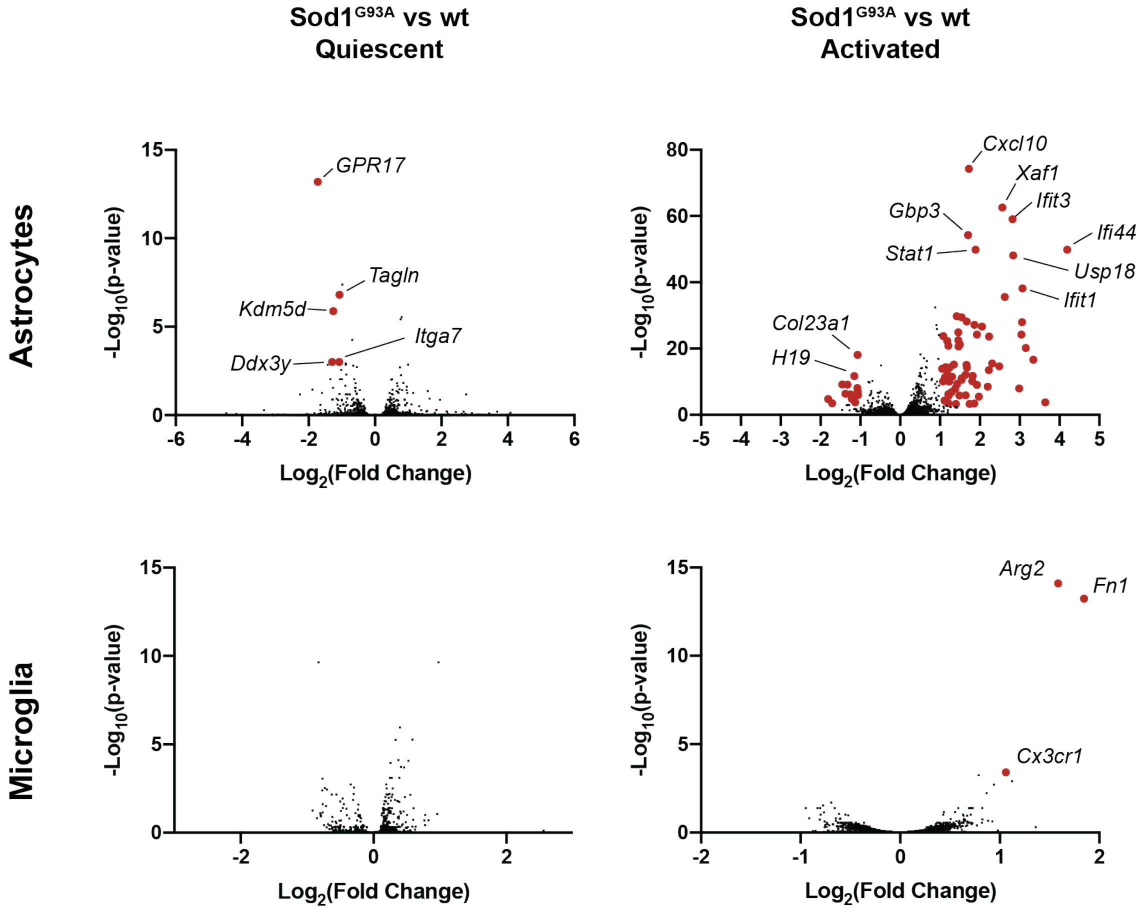

Supplementary Figure 4. Detailed analysis of in vitro Sod1^{G93A} vs wt astrocytes and microglia when quiescent and activated. Data from Figure 2a-b,e-f re-graphed and partially annotated to show details of the slight differences between *Sod1^{G93A}* and WT microglia when quiescent and the larger differences between *Sod1^{G93A}* and WT astrocytes when quiescent and activated.

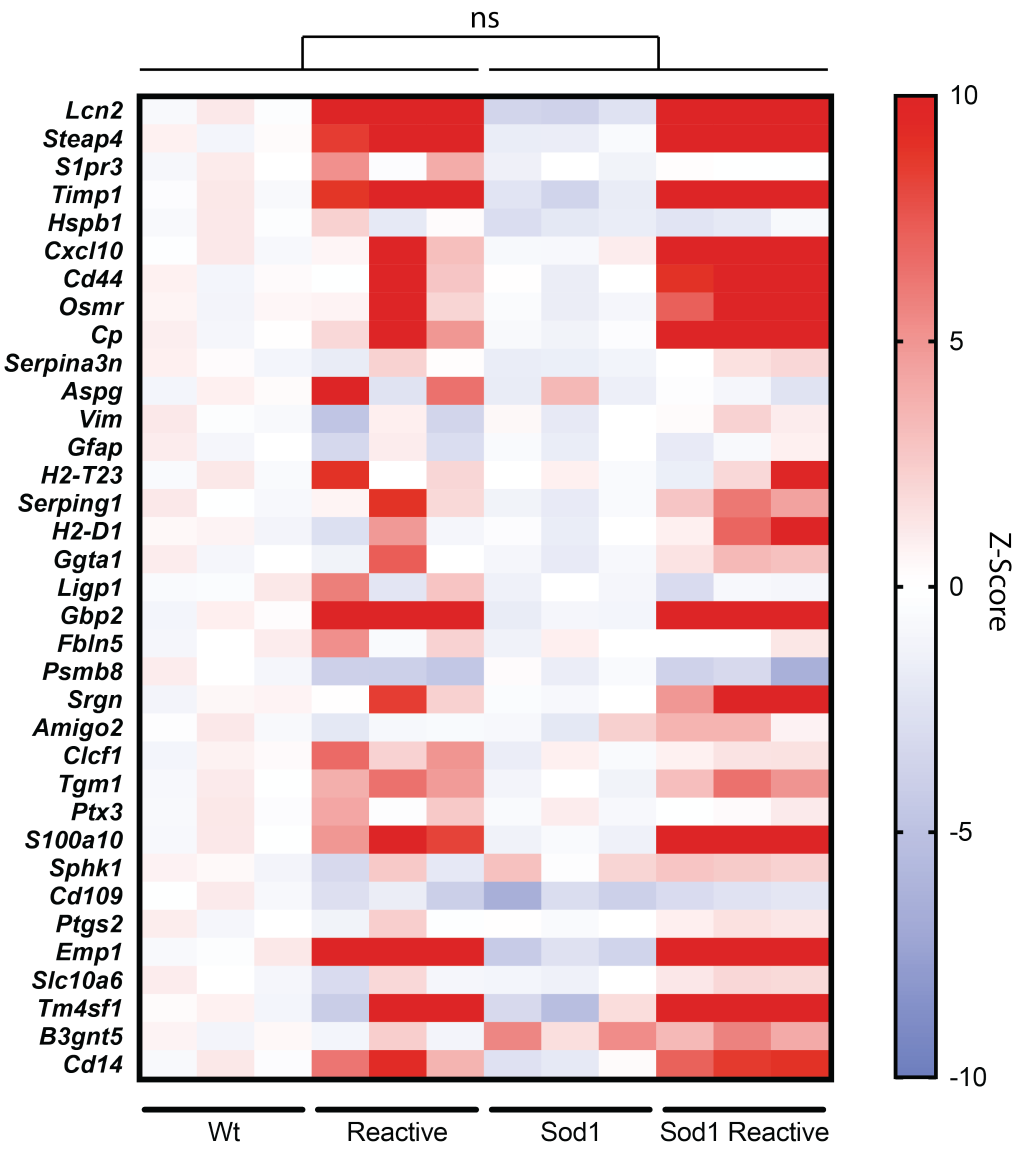

Supplementary Figure 5. *In vitro* astrocytes show similar reactivity signature when fully activated. We performed microfluidics qPCR on *in vitro* $Sod1^{G93A}$ and WT astrocytes grown in serum-free conditions to assess astrocyte reactivity subtype. The upregulation of astrocyte reactivity genes in response to maximum stimulation by IL-1α, TNFα, and C1q was compared between $Sod1^{G93A}$ and WT cells. No significant difference in activation markers was found (two-tailed, unpaired Student's t-tests, corrected by Holm-Sidak method). Because we saw no difference in these markers in response to maximal activation, to assess the activation of astrocytes to subthreshold doses of IL-1α, TNFα, and C1q we normalized the response of each gene to its maximum change in response to full activation.

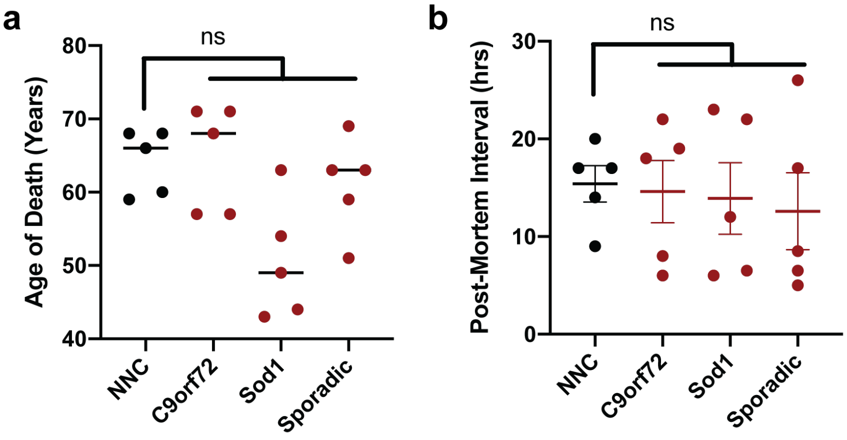

Supplementary Figure 6. Human tissue samples are not significantly different in age of death or post-mortem interval of tissue collection. a. Age of death is not significantly different between groups of human patient tissue. (lines represent means) **b.** Post-mortem interval between death and tissue collection is not significantly different between groups of human patient tissue. (NNC = non-neurological control; lines represent mean ± SEM; ns = p > 0.05 by Kruskal-Wallis non-parametric test corrected for multiple comparisons using the Dunn method)

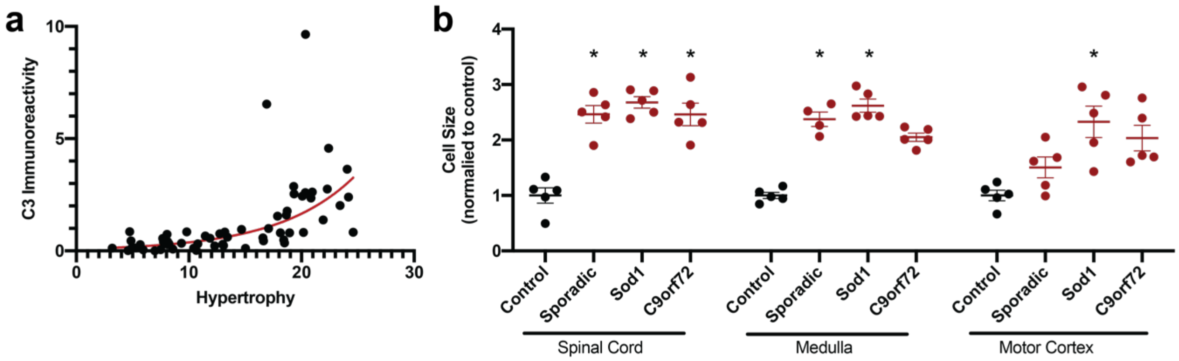

Supplementary Figure 7. Relationship between C3 expression and hypertrophy in astrocytes from human tissue. a. Comparing C3 immunoreactivity to percent GFAP$^+$ area in human tissue sections as a measure of cell body and process hypertrophy in individual images shows a positive relationship between the measures. Red line represents an exponential growth curve fit to the data. **b.** Percent GFAP$^+$ area in human tissues sections as a measure of hypertrophy normalized to control conditions shows significant astrocyte hypertrophy in many ALS patient conditions, especially in those areas with greater increases in C3$^+$ immunoreactivity (Figure 3) (lines represent mean ± SEM; * = p < 0.05 by Kruskal-Wallis non-parametric test corrected for multiple comparisons using the Dunn method; mean ± s.e.m.).

Go Term	Fold Enrichment	FDR
**positive regulation of interferon-gamma-mediated signaling pathway**	> 100	8.46E-05
**defense response to protozoan**	59.51	5.18E-09
**adhesion of symbiont to host**	51.39	6.48E-04
**positive regulation of interferon-alpha production**	39.26	1.24E-04
**cytoplasmic pattern recognition receptor signaling pathway**	35.33	2.58E-02

Supplementary Table 1. Go Term analysis of Sod^{G93A} reactive astrocyte genes. The 77 genes upregulated in activated Sod1^{G93A} astrocytes vs wt activated astrocytes were analyzed by go-term analysis relative to all genes expressed in astrocytes using the Go Biological Process Complete annotation through Panther DB. Reported here are the 5 unique Go Terms with highest Fold Enrichment (manually compiled).

Sample Group	Sex	Age of Death	PMI (h)
unremarkable adult brain	Male	59	17
unremarkable adult brain	Male	68	14
unremarkable adult brain	Male	66	20
unremarkable adult brain	Female	60	9
unremarkable adult brain	Male	68	17
C9orf72 expansion	Male	57	6
C9orf72 expansion	Male	71	18
C9orf72 expansion	Male	57	22
C9orf72 expansion	Female	71	19
C9orf72 expansion	Female	68	8
SOD1 c.374a>T,p.D125V	Female	63	6.5
SOD1 c.14C>T,p.A5V	Female	54	6
SOD1 c.13G>A,p.A5T	Male	49	22
SOD1 c.281G>C,p.G94A	Female	43	12
SOD1 p.His47Asp	Male	44	23
sporadic	Male	51	17
sporadic	Male	63	5
sporadic	Male	63	6.5
sporadic	Female	59	26
sporadic	Male	69	8.5

Supplementary Table 2. Clinical characteristics of human post-mortem samples from ALS patients and non-neurological controls. Basic clinical information for post-mortem samples.

Figure	Condition	n
1b	wt lumbar	6
	Sod1G93A (80-110d) lumbar	9
	Sod1G93A (125-150d) lumbar	9
	Il1a-/-TNFα-/-C1q-/-Sod1G93A (190-210d) lumbar	8
	wt thoracic	6
	Sod1G93A (80-110d) thoracic	9
	Sod1G93A (125-150d) thoracic	9
	Il1a-/-TNFα-/-C1q-/-Sod1G93A (125-150d) thoracic	9
	wt cervical	6
	Sod1G93A (80-110d) cervical	8
	Sod1G93A (125-150d) cervical	7
	Il1a-/-TNFα-/-C1q-/-Sod1G93A (125-150d) cervical	9
	wt medulla	3
	Sod1G93A (80-110d) medulla	3
	Sod1G93A (125-150d) medulla	3
	Il1a-/-TNFα-/-C1q-/-Sod1G93A (125-150d) medulla	3
	wt cortex	3
	Sod1G93A (80-110d) cortex	3
	Sod1G93A (125-150d) cortex	3
	Il1a-/-TNFα-/-C1q-/-Sod1G93A (125-150d) cortex	3
1c	Sod1G93A	25
	Il1a-/-TNFα-/-C1q-/-Sod1G93A	19
1d	wt	11
	Sod1G93A	19
	Il1a-/-TNFα-/-C1q-/-	12
	Il1a-/-TNFα-/-C1q-/-Sod1G93A	19
1e	wt	10
	Sod1G93A	8
	Il1a-/-TNFα-/-C1q-/-	7
	Il1a-/-TNFα-/-C1q-/-Sod1G93A	10
1g	wt	4
	Sod1G93A	21
	Il1a-/-TNFα-/-C1q-/-	5
	Il1a-/-TNFα-/-C1q-/-Sod1G93A	26

Figure	Condition	n
1i	wt (125-150d) lumbar	6
	Sod1G93A (125-150d) lumbar	9
	Il1a-/-TNFα-/-C1q-/- (190-210d) lumbar	11
	Il1a-/-TNFα-/-C1q-/-Sod1G93A (190-210d) lumbar	8
	wt (125-150d) thoracic	6
	Sod1G93A (125-150d) thoracic	10
	Il1a-/-TNFα-/-C1q-/- (190-210d) thoracic	5
	Il1a-/-TNFα-/-C1q-/-Sod1G93A (190-210d) thoracic	9
	wt (125-150d) cervical	6
	Sod1G93A (125-150d) cervical	9
	Il1a-/-TNFα-/-C1q-/- (190-210d) cervical	5
	Il1a-/-TNFα-/-C1q-/-Sod1G93A (190-210d) cervical	6
2	Each genotype in each experiment	3
3b	Control Spinal Cord	5
	Sporadic Spinal Cord	5
	Sod1 Spinal Cord	5
	C9orf72 Spinal Cord	5
	Control Medulla	5
	Sporadic Medulla	5
	Sod1 Medulla	4
	C9orf72 Medulla	5
	Control Motor Cortex	5
	Sporadic Motor Cortex	5
	Sod1 Motor Cortex	5
	C9orf72 Motor Cortex	5
3c	Non-Neurological Control	16
	ALS	148
3d	Non-Neurological Control	16
	C9orf72	15
	Sporadic	20
	Sod1	5
	Other Genetic	22

Supplementary Table 3. Technical Details of Experiments. Number of biological replicates used in each experiment in the main figures of the manuscript.

Figure	Comparison		P Value (corrected, if applicable)
1b	Medulla Sod1G93A vs wt		0.0239
	Medulla Il1a-/-TNFa-/-C1q-/-Sod1G93A vs Sod1G93A		0.0236
	Cervical Sod1G93A vs wt		0.0007
	Cervical Il1a-/-TNFa-/-C1q-/-Sod1G93A vs Sod1G93A		0.0006
	Thoracic Sod1G93A vs wt		0.0239
	Thoracic Il1a-/-TNFa-/-C1q-/-Sod1G93A vs Sod1G93A		0.0091
	Lumbar Sod1G93A vs wt		0.0007
	Lumbar Il1a-/-TNFa-/-C1q-/-Sod1G93A vs Sod1G93A		0.0006
1c	Il1a-/-TNFa-/-C1q-/-Sod1G93A vs Sod1G93A		<0.0001
1d	Time (weeks)	Sod1G93A vs wt	Il1a-/-TNFa-/-C1q-/-Sod1G93A vs Il1a-/-TNFa-/-C1q-/-
	8	0.8694	0.9387
	9	0.2687	0.9173
	10	0.1997	0.1712
	11	0.0039	0.3245
	12	0.0003	0.0321
	13	<0.0001	0.0141
	14	<0.0001	0.014
	15	<0.0001	<0.0001
	16	<0.0001	<0.0001
	17	<0.0001	<0.0001
	18	<0.0001	<0.0001
	19	<0.0001	<0.0001
	20	<0.0001	<0.0001
	21	<0.0001	<0.0001
	22	<0.0001	<0.0001
	23	<0.0001	<0.0001
	24	<0.0001	<0.0001
	25	<0.0001	<0.0001
	26	<0.0001	<0.0001
	27	<0.0001	<0.0001
	28	<0.0001	<0.0001
	29	0.0054	<0.0001
	30	<0.0001	<0.0001
1i	Cervical Sod1G93A (125-150d) vs wt		0.1108
	Cervical Il-1a-/- TNFa-/- C1q-/- Sod1G93A (125-150d) vs wt		0.9968
	Cervical Il-1a-/- TNFa-/- C1q-/- Sod1G93A (190-210d) vs wt		0.9951
	Thoracic Sod1G93A (125-150d) vs wt		0.8310
	Thoracic Il-1a-/- TNFa-/- C1q-/- Sod1G93A (125-150d) vs wt		0.9997
	Thoracic Il-1a-/- TNFa-/- C1q-/- Sod1G93A (190-210d) vs wt		0.9961
	Lumbar Sod1G93A (125-150d) vs wt		0.0032
	Lumbar Il-1a-/- TNFa-/- C1q-/- Sod1G93A (125-150d) vs wt		>0.9999
	Lumbar Il-1a-/- TNFa-/- C1q-/- Sod1G93A (190-210d) vs wt		0.1177
	Lumbar Il-1a-/- TNFa-/- C1q-/- Sod1G93A (190-210d) vs Sod1G93A (125-150d)		0.9888
2h	TNFa Sod1 vs wt 0 ng/ml		0.988753
	TNFa Sod1 vs wt 0.5 ng/ml		0.046320
	TNFa Sod1 vs wt 1 ng/ml		0.002126
	TNFa Sod1 vs wt 5 ng/ml		<0.000001
	Il-1a Sod1 vs wt 0 ng/ml		>0.999999
	Il-1a Sod1 vs wt 0.5 ng/ml		0.000290
	Il-1a Sod1 vs wt 1 ng/ml		0.000004
	Il-1a Sod1 vs wt 5 ng/ml		0.000290
	CCL2 Sod1 vs wt 0 ng/ml		0.963046
	CCL2 Sod1 vs wt 0.5 ng/ml		0.022432
	CCL2 Sod1 vs wt 1 ng/ml		0.000084
	CCL2 Sod1 vs wt 5 ng/ml		0.000122
	Il-6 Sod1 vs wt 0 ng/ml		0.958231
	Il-6 Sod1 vs wt 0.5 ng/ml		0.007008
	Il-6 Sod1 vs wt 1 ng/ml		0.097128
	Il-6 Sod1 vs wt 5 ng/ml		0.085450
	Cd69 Sod1 vs wt 0 ng/ml		>0.999999
	Cd69 Sod1 vs wt 0.5 ng/ml		0.000063
	Cd69 Sod1 vs wt 1 ng/ml		0.046054
	Cd69 Sod1 vs wt 5 ng/ml		0.046054
	Nos2 Sod1 vs wt 0 ng/ml		0.980860
	Nos2 Sod1 vs wt 0.5 ng/ml		0.496032
	Nos2 Sod1 vs wt 1 ng/ml		0.044997

	Nos2 Sod1 vs wt 5 ng/ml	<0.000001
**3b**	Spinal Cord Sporadic vs Control	0.0485
	Spinal Cord Sod1 vs Control	0.0117
	Spinal Cord C9orf72 vs Control	0.0192
	Medulla Sporadic vs Control	0.0022
	Medulla Sod1 vs Control	0.0066
	Medulla C9orf72 vs Control	0.5886
	Motor Cortex Sporadic vs Control	0.0099
	Motor Cortex Sod1 vs Control	0.0309
	Motor Cortex C9orf72 vs Control	0.036
**3c**	ALS vs Control	<0.0001
**3d**	C9orf72 vs Control	<0.0001
	Sporadic vs Control	0.0017
	Sod1 vs Control	<0.0001
	Other Genetic vs Control	<0.0001

Supplementary Table 4. Statistical Details of Experiments. Exact p-values for statistical comparisons in main figures.

Gene	Forward	Reverse
Lcn2	CCGACACTGACTACGACCAG	AATGCATTGGTCGGTGGGAA
Steap4	CAAACGCCGAGTACCTTGCT	CAGACAAACACCTGCCGACT
S1pr3	CTTGCAGAACGAGAGCCTGT	CCTCAACAGTCCACGAGAGG
Timp1	CGCTAGAGCAGATACCACGA	CCAGGTCCGAGTTGCAGAAA
Hspb1	GAGATCACTGGCAAGCACGA	ATTGTGTGACTGCTTTGGGC
Cxcl10	TGCAAGTCTATCCTGTCCGC	ACGGAGCTCTTTTTGACCTTC
Cd44	TCAGGATAGCCCCACAACAAC	GACTCCGTACCAGGCATCTTC
Osmr	GTCATTCTGGACATGAAGAGGT	AATCACAGCGTTGGGTCTGA
Cp	GATGTTTCCCCAAACGCCTG	GTAGCTCTGAGACGATGCTTGA
Serpina3n	GTCTTTCAGGTGGTCCACAAGG	GCCAATCACAGCATAGAAGCG
Aspg	CAGGTGCCCAGGTTCCTATC	GTCCACCTTGGTTGTCCGAT
Vim	GAGGAGATGAGGGAGTTGCG	CTGCAATTTTTCTCGCAGCC
Gfap	AACCGCATCACCATTCCTGT	TCCTTAATGACCTCGCCATCC
H2-T23	ATTGGAGCTGTTGTGAGGAGG	CCACGAGGCAACTGTCTTTTC
Serping1	TGGCTCAGAGGCTAACTGGC	GAATCTGAGAAGGCTCTATCCCCA
H2-D1	ATGGAACCTTCCAGAAGTGGG	GAAGTAAGTTGGAGTCGGTGGA
Ggta1	TCTCAGGATCTGGGAGTTGGA	GAGTTCTATGGAGCTCCCGC
Ligp1	ATTTGGCTCGAAGCCTTTGC	ACGGCATTTGCCAGTCCTTA
Gbp2	TAAAGGTCCGAGGCCCAAAC	AACATATGTGGCTGGGCGAA
Fbln5	AGGGGGTTAAGCGAAACCAG	GTGAGTATCCTTTTAATCCTGGCA
Psmb8	TATCTGCGGAATGGGGAACG	AAAGTCCCGGTCCCTTCTTG
Srgn	GTTCAAGGTTATCCTGCTCGGA	AAACAGGATCGGTCATCGGG
Amigo2	GTTCGCCACAACAACATCAC	GTTTCTGCAAGTGGGAGAGC
Clcf1	GACTCGTGGGGGATGTTAGC	CCCCAGGTAGTTCAGGTAGGT
Tgm1	AGACCCAATTTTCCTGGGGC	AGCGAGGACCTTCCATTGTG
Ptx3	CATCCCGTTCAGGCTTTGGA	CACAGGGAAAGAAGCGAGGT
S100a10	GAAAGGGAGTTCCCTGGGTT	CCCACTTTTCCATCTCGGCA
Sphk1	AAAGCGAGACCCTGTTCCAG	CAGTCTGCTGGTTGCATAGC
Cd109	GTCGCTCACAGGTACCTCAA	CTGTGAAGTTGAGCGTTGGC
Ptgs2	CTCAGCCATGCAGCAAATCC	GGGTGGGCTTCAGCAGTAAT
Emp1	ACCATTGCCAACGTCTGGAT	TGGAACACGAAGACCACGAG
Slc10a6	TCCATAGAGACCGGAGCACA	ATGCCTGATATGCTGCGACA
Tm4sf1	CTGAGGGACAGTACCTTCTGGATTC	GGCTAGGCCTCAACACAGTTA
B3gnt5	TGCTCCTGGATGAAAGGTCC	ACATGCTTGATCCGTGTGGT
Cd14	TCAGAATCTACCGACCATGAAGC	GGACACTTTCCTCGTCCTGG

Supplementary Table 5. Primers used in Supplementary Figure 5. Primers used for microfluidics qPCR quantification of mouse astrocyte reactivity.
