# Supplementary material for: Knockout of reactive astrocyte activating factors slows disease progression in an ALS mouse model
Source: Nat Commun. 2020 Jul 27;11:3753. doi: 10.1038/s41467-020-17514-9 (PMC7385161; doi:10.1038/s41467-020-17514-9)
Supplement: Supplementary file 3 — Reporting Summary [file 41467_2020_17514_MOESM3_ESM.pdf]

natureresearch

Corresponding author(s): Kevin Guttenplan
Aaron Gitler

Last updated by author(s): Jun 20, 2020

Reporting Summary

Nature Research wishes to improve the reproducibility of the work that we publish. This form provides structure for consistency and transparency in reporting. For further information on Nature Research policies, see Authors & Referees and the Editorial Policy Checklist .

Statistics

For all statistical analyses, confirm that the following items are present in the figure legend, table legend, main text, or Methods section.

n/a	Confirmed	
☐	☒	The exact sample size (*n*) for each experimental group/condition, given as a discrete number and unit of measurement
☐	☒	A statement on whether measurements were taken from distinct samples or whether the same sample was measured repeatedly
☐	☒	The statistical test(s) used AND whether they are one- or two-sided<br>*Only common tests should be described solely by name; describe more complex techniques in the Methods section.*
☒	☐	A description of all covariates tested
☐	☒	A description of any assumptions or corrections, such as tests of normality and adjustment for multiple comparisons
☐	☒	A full description of the statistical parameters including central tendency (e.g. means) or other basic estimates (e.g. regression coefficient) AND variation (e.g. standard deviation) or associated estimates of uncertainty (e.g. confidence intervals)
☐	☒	For null hypothesis testing, the test statistic (e.g. *F*, *t*, *r*) with confidence intervals, effect sizes, degrees of freedom and *P* value noted<br>*Give P values as exact values whenever suitable.*
☒	☐	For Bayesian analysis, information on the choice of priors and Markov chain Monte Carlo settings
☒	☐	For hierarchical and complex designs, identification of the appropriate level for tests and full reporting of outcomes
☒	☐	Estimates of effect sizes (e.g. Cohen's *d*, Pearson's *r*), indicating how they were calculated

Our web collection on statistics for biologists contains articles on many of the points above.

Software and code

Policy information about availability of computer code

Data collection	Microfluidics qPCR: BioMark Data Collection Software 2.1.1 build 20090519.0926; Confocal Imaging: Zen 2012 v. 14.09.201
Data analysis	RNAseq: STAR v2.7.3a, R v3.6.1, DESeq2 package v1.38.0; Microfluidics qPCR: BioMark Data Collection Software 2.1.1 build 20090519.0926, Melting Curve Analysis Software 1.1.0 build 20100514.1234, Real-time PCR Analysis Software 2.1.1 build 20090521.1135; Statistics - Prism v 8.2.1; Image Analysis - ImageJ v2.0.0 build 269a0ad53f; RNA sequencing pipeline available at https://github.com/emc2cube/Bioinformatics/

For manuscripts utilizing custom algorithms or software that are central to the research but not yet described in published literature, software must be made available to editors/reviewers. We strongly encourage code deposition in a community repository (e.g. GitHub). See the Nature Research guidelines for submitting code & software for further information.

Data

Policy information about availability of data

All manuscripts must include a data availability statement. This statement should provide the following information, where applicable:
- Accession codes, unique identifiers, or web links for publicly available datasets
- A list of figures that have associated raw data
- A description of any restrictions on data availability

In vitro RNAseq data available at website www.gliaseq.com. Raw in vitro RNAseq data available at https://www.ncbi.nlm.nih.gov/geo/query/acc.cgi?acc=GSE143598. All other data are available from the corresponding author upon reasonable request.

Field-specific reporting

Please select the one below that is the best fit for your research. If you are not sure, read the appropriate sections before making your selection.

☒ Life sciences ☐ Behavioural & social sciences ☐ Ecological, evolutionary & environmental sciences

For a reference copy of the document with all sections, see nature.com/documents/nr-reporting-summary-flat.pdf

Life sciences study design

All studies must disclose on these points even when the disclosure is negative.

Sample size	Sample size was determined by prior literature using similar experimental approaches rather than by power analysis. Sample sizes for in vivo characterization of Sod1G93A mice was based on previous studies of large-effect manipulations of this mouse (Kostic et al., Science, 1997) as well as in accordance with guidelines set for the study of this mouse model (Scott et al., Design, power, and interpretation of studies in the standard murine model of ALS, Amyotrophic Lateral Sclerosis, 2008). n for in vitro studies and pathological sample studies were based on previous experiments manipulating reactivity in primary glial cells as well as staining for C3 in human tissue (Liddelow et al., 2017).
Data exclusions	The medulla tissue samples of one Sod1 ALS patient was excluded after several staining attempts failed to achieve specific GFAP staining of astrocytes and thus prevented the quantification of C3 immunofluorescence within astrocytes, an exclusion criteria established before experiment onset. No other data was excluded.
Replication	All animal studies were performed on animals from different litters over many months. Primary cell culture was performed on cells isolated from at least 2 unique animal preparations. Tissue staining experiments were performed 3 times over the course of several months. All attempts at replication were successful.
Randomization	Mice were randomly selected within genotype for assignment to different experiments. All mouse experiments were performed by comparing genotypes and thus randomization within mouse experiment type was not relevant as there were not different treatment groups within genotype within one experimental type. For in vitro studies, wells were randomly selected for dose of activator.
Blinding	All mice were given a number after birth and subsequent experiments performed blind to age and genotype. Selection of NMJs for confocal analysis was based on alpha-bungarotoxin signal blind to age, genotype, and NF signal. Subsequent analysis of NMJ innervation was performed blind to age and genotype. Imaging of human ALS slides was performed on the basis of GFAP staining blind to patient information and C3 signal. Motor neurons were imaged and counted blinded to age and genotype. RNA libraries were prepared by an Agilent Bravo Automated Liquid Handling Platform that was blind to genotype and condition. All in vitro experiments were performed blind to cell genotype and treatment.

Reporting for specific materials, systems and methods

We require information from authors about some types of materials, experimental systems and methods used in many studies. Here, indicate whether each material, system or method listed is relevant to your study. If you are not sure if a list item applies to your research, read the appropriate section before selecting a response.

Materials & experimental systems

n/a	Involved in the study	
☐	☒	Antibodies
☒	☐	Eukaryotic cell lines
☒	☐	Palaeontology
☐	☒	Animals and other organisms
☐	☒	Human research participants
☒	☐	Clinical data

Methods

n/a	Involved in the study	
☒	☐	ChIP-seq
☒	☐	Flow cytometry
☒	☐	MRI-based neuroimaging

Antibodies

Antibodies used	Alpha-Bungarotoxin (Invitrogen, B35450); Rb anti NF (abcam ab8135); Rb anti C3d (Dako, A0063); Ms anti GFAP (Sigma G3893); Alexa-594 goat anti-rabbit (ThermoFisher R37117); Alexa-647 goat anti-mouse (ThermoFisher A-21235)
Validation	Alpha-Bungarotoxin - see https://doi.org/10.1016/B978-0-12-185266-5.50011-7 ; Rb anti NF - see https://www.abcam.com/neurofilament-heavy-polypeptide-antibody-ab8135.html#description_images_6 ; Rb anti C3d - see https://www.agilent.com/cs/library/packageinsert/public/102272002.PDF ; Ms anti GFAP - see https://www.sigmaaldrich.com/content/dam/sigma-aldrich/docs/Sigma/Datasheet/7/g3893dat.pdf; Alexa-594 goat anti-rabbit - see https://www.thermofisher.com/order/genome-database/dataSheetPdf?producttype=antibody&productsubtype=antibody_secondary&productId=R37117&version=105; Alexa-647 goat anti-mouse - see https://www.thermofisher.com/order/genome-database/dataSheetPdf?producttype=antibody&productsubtype=antibody_secondary&productId=A-21235&version=105

Animals and other organisms

Policy information about studies involving animals; ARRIVE guidelines recommended for reporting animal research

Laboratory animals	All mice were housed with food and water available ad libitum in a 12-h light/dark environment at 68-72F and 30-70% humidity. IL-1-/- TNF-/- C1qa-/- animals were developed in house (Liddelow et al., Nature, 2016). Sod1G93A were obtained from Jax (002726) and bred into either a C57BL6 (Jax, 000664) or IL-1-/- TNF-/- C1q-/- C57BL6 line. Mixed sex mice were used in all studies. Glial cell isolation was performed in p5-p10 mice. In vivo experiments were performed in ages ranging from P30 to P300.
Wild animals	This study did not involve wild animals.
Field-collected samples	This study did not involve samples collected from the field.
Ethics oversight	Administrative Panel of Laboratory Animal Care (APLAC) of Stanford University, an institution accredited by the Association for the Assessment and Accreditation of Laboratory Animal Care (AAALAC)

Note that full information on the approval of the study protocol must also be provided in the manuscript.

Human research participants

Policy information about studies involving human research participants

Population characteristics	Tissue obtained from the University of Pennsylvania Alzheimer's Disease Core Center (ADCC) Neuropathology, Genetics and Biomarker Core. Tissue was fixed and de-identified before acquisition.
Recruitment	N/A
Ethics oversight	The Federalwide Assurance (FWA) for NYU Langone (#00004952) was approved by the Office for Human Research Protections at the U.S. Department of Health and Human Services (DHHS). All tissue was de-identified and provided without link to identifiable information from an IRB-approved tissue repository.

Note that full information on the approval of the study protocol must also be provided in the manuscript.
