# Supplementary material for: Knockout of reactive astrocyte activating factors slows disease progression in an ALS mouse model
Source: Nat Commun. 2020 Jul 27;11:3753. doi: 10.1038/s41467-020-17514-9 (PMC7385161; doi:10.1038/s41467-020-17514-9)
Supplement: Supplementary file 2 — Peer Review File [file 41467_2020_17514_MOESM2_ESM.pdf]

Editorial Note: This manuscript has been previously reviewed at another journal that is not operating a transparent peer review scheme. The manuscript was considered suitable for publication without further review at *Nature Communications.*
